# The Influence of Body Mass Index, Age and Sex on Inflammatory Disease Risk in Semi-Captive Chimpanzees

**DOI:** 10.1371/journal.pone.0104602

**Published:** 2014-08-14

**Authors:** Vincent Obanda, George Paul Omondi, Patrick Ilukol Chiyo

**Affiliations:** 1 Veterinary Services Department, Kenya Wildlife Service, Nairobi, Kenya; 2 Faculty of Veterinary Medicine, University of Nairobi, Nairobi, Kenya; 3 Ol Pejeta Conservancy, Private Bag, Nanyuki, Kenya; 4 Department of Biological Sciences, University of Notre Dame, Notre Dame, Indiana, United States of America; 5 Department of Biology, Duke University, Durham, North Carolina, United States of America; Institut Pluridisciplinaire Hubert Curien, France

## Abstract

Obesity and ageing are emerging issues in the management of captive primates, including Chimpanzees, *Pan troglodytes*. Studies on humans show that obesity and old age can independently increase the risk of inflammatory-associated diseases indicated by elevated levels of pro-inflammatory cells and proteins in the blood of older or obese compared to levels in younger or non-obese individuals. In humans, sex can influence the outcomes of these risks. Health management of these problems in chimpanzee populations requires an understanding of similarities and differences of factors influencing inflammatory disease risks in humans and in chimpanzees. We examined the relationship between age, sex and Body Mass Index (BMI) with hematological biomarkers of inflammatory disease risk established for humans which include the neutrophil to lymphocyte ratio (NLR), and neutrophil, white blood cell (WBC), platelet microparticle and platelet counts. We found that higher values of NLR, neutrophil count and platelet microparticle count were associated with higher BMI values and older age indicating increased inflammation risk in these groups; a similar pattern to humans. There was a strong sex by age interaction on inflammation risk, with older males more at risk than older females. In contrast to human studies, total WBC count was not influenced by BMI, but like humans, WBC and platelet counts were lower in older individuals compared to younger individuals. Our findings are similar to those of humans and suggest that further insight on managing chimpanzees can be gained from extensive studies of ageing and obesity in humans. We suggest that managing BMI should be an integral part of health management in captive chimpanzee populations in order to partially reduce the risk of diseases associated with inflammation. These results also highlight parallels in inflammation risk between humans and chimpanzees and have implications for understanding the evolution of inflammation related diseases in apes.

## Introduction

Obesity and ageing are emerging issues in the management of captive primates including macaques [1]–[3], lemurs [4], [5] and chimpanzees [6]–[8] because of increased morbidity and mortality related to obesity and old age in captivity. Studies on humans show that obesity and old age can independently increase the risk of inflammatory-associated diseases [9], [10] and the outcomes of these risks are different for males and females [11], [12]. Inflammation is an immunological response to tissue injury or infection involving several cellular and biochemical processes that destroy harmful agents, liquefy damaged tissues and rebuild new tissues [13], [14]. Although inflammation is a normal homeostatic process, that is often temporary and localized to areas of infection or injury, chronic and systemic inflammation signals dis-regulation or persistent activation of the pro-inflammatory response [15].

Age-associated decline of immune function due to redox imbalance, obesity, social stress, and chronic infections by pathogens are some of the factors that induce persistent dysregulation and activation of the pro-inflammatory response [15]–[23]. For example, low-grade, chronic inflammation is mediated by increased levels of pro-inflammatory proteins due to an age-related redox imbalance which stimulates many pro-inflammatory signaling pathways [16], [24]. Blood levels of pro-inflammatory proteins are known to increase with advancing age [12], [25], [26]. Several studies have shown that chronic inflammation is a major risk factor underlying aging and age-related diseases [27].

The incidence of inflammatory disease risk is also higher in heavy-weight and obese individuals than lean individuals independent of age [28]–[30]. Obesity is known to induce a chronic baseline state of inflammation through increased expression of pro-inflammatory proteins in adipose tissue [22], [31]–[35]. There is also increased recruitment of macrophages in adipose tissue of obese individuals compared to lean individuals [36]. Macrophages exacerbate the inflammation state by producing pro-inflammatory cytokines including, Tissue Necrotic Factor- α, InterLeukin-6, and inducible Nitric Oxide Synthase.

Sex has a major influence on immune response to infection and injury. Females usually have a more elevated pro-inflammation state following infection or injury than males [28], [29], [37]–[41]. Contrary to the elevated pro-inflammatory state in females following infection or injury, males incur more mortality from inflammatory related diseases than females. This discrepancy in mortality arises because females are often protected from inflammation related diseases by sex hormones such as estradiol [11], [42].

Increase in baseline pro-inflammatory state is thought to play a major role in the progression and pathogenesis of many human diseases including atherosclerosis and cardiovascular disease (e.g. cardiac fibrosis, cardiomyopathy, coronary heart disease, myocarditis), cystic fibrosis, asthma, osteoporosis, inflammatory bowel disease, diabetes, psoriasis, and rheumatoid arthritis [10], [43], [44]. Cardiovascular disease is the leading cause of human mortality in the developed countries and the disease burden is rapidly growing in developing countries [45]. The incidence of cardiovascular disease is higher in older ages in both sexes, but the incidence is elevated in males compared to females [42], [46].

Like humans, cardiovascular disease, specifically cardiac fibrosis, is a major cause of mortality in captive chimpanzees, *Pan troglodytes* [47]–[50]. Mortality from cardiovascular disease is higher in older individuals compared to younger individuals and in males compared to females [49]. Female chimpanzees with heavy weights for their age are more likely than light-weight females to develop and die from cardiovascular disease [49]. These mortality patterns are similar to those caused by cardiovascular diseases in humans [42], [51], [52]. Although age, obesity and sex are known to influence the baseline level of inflammation and to predict the risk of mortality from inflammatory diseases such as cardiovascular disease in humans, not much is known regarding the correlation between age, BMI, sex and cellular hematological biomarkers of inflammation in chimpanzees.

Many cellular components used as biomarkers of inflammation and inflammatory disease risk play a role in inflammation. For example neutrophils, a component of WBCs, are usually the first cells to populate injured or infected tissue where they initiate a short-lived inflammatory response. Neutrophils kill microbes by releasing of microbicidal peptides or proteolytic enzymes (e.g. α-defensins, serine protease, elastase, and lysozyme) and Reactive Oxygen Species or ROS (e.g. hydrogen peroxide, hypochlorus acid, hydroxyl radical, and chloramines) into infected cells. However, chronically elevated levels of neutrophils can occur -as a result of dis-regulation of neutrophil apoptosis due to chronic infection or oxidative stress- causing collateral tissue damage [53], [54]. This condition will result in a high Neutrophil-lymphocyte ratio (NLR), which is negatively correlated with anti-inflammatory high-density lipoprotein cholesterol [55]. NLR is used as a measure of inflammatory disease risk and to predict the probability of developing inflammatory disease [56]–[63].

Platelets are another group of cells that play a critical role during inflammation. Once activated, platelets release microparticles, growth factors, chemokines, cytokines (e.g. tissue necrotic factor-α, interleukin-1, interleukin-6 and interleukin-8) that help maintain and modulate inflammation and play a role in fibrinolysis, wound healing and angiogenesis [64], [65]. High levels of pro-inflammatory cytokines such as, interleukin-1 (IL-1), interleukin-6 (IL-6) and Tissue Necrotic Factor-α (TNF-α) are known to stimulate the release of acute-phase proteins such as C-Reactive Proteins, serum amyloid-A, and fibrinogen by liver cells [66], [67]. These acute phase proteins and their predecessor cytokines are used as markers of inflammation.

Platelet microparticle (µPLT) counts are tightly correlated with pro-inflammatory proteins and cytokines including IL-6, C-Reactive Proteins, and serum amyloid-A which are often used as standard biomarkers of inflammation risk [68]. µPLT counts have recently been used as biomarkers of inflammation because individuals with inflammatory disease have an elevated count of platelet microparticles compared to healthy individuals [69]. Elevated counts of µPLT have also been shown to predict future risk of morbidity and mortality from inflammatory diseases [69], [70].

In this study, we examined the correlations of each hematological biomarker of inflammation with age, sex and BMI in chimpanzees. First, we examined biomarkers of inflammation established for humans (i.e. NLR and the proportion of µPLT from the total platelet component of the blood) for their correlations with chimpanzee attributes such as BMI, age and sex. We predicted that NLR and the proportion of µPLT will be positively correlated with age and BMI. We also predicted that NLR and µPLT will be higher in males compared to females. Second, we examined the relationships between individual animals traits (age, sex and BMI) and cell components that are sometimes correlated with inflammation [71], [72] -including total WBC and platelet counts and the proportion WBC that are neutrophils or lymphocytes. We predicted that neutrophils, total WBC and platelets will be more elevated in animals with a higher BMI than in animals with lower BMI. Neutrophils will be elevated in older animals than in younger animals, but total WBC and platelets will decline with age. We also predicted that males will have higher total platelet and WBC counts than females. Third, we examined the influence of age, BMI and sex on counts of red blood cell (RBC) and RBC microparticles (µRBC). RBCs and µRBCs are not known to be influenced by inflammation, but their quantities may be influenced independently by age and sex. We therefore predicted that these non-inflammatory blood components will be correlated with age and sex but not with BMI.

## Materials and Methods

### Ethics Statement

The data used in this study was obtained during a health review of chimpanzees of the Sweetwaters Chimpanzee Sanctuary (SCS). This health review was conducted as part of routine care for this population and involved physical examination as well as hematological and biochemical analyses of blood and blood serum respectively. Guidelines for these health reviews were approved by the Sweetwaters Chimpanzee Sanctuary Steering Committee, Kenya Wildlife Service and the Pan-African Sanctuaries Alliance (PASA). Regular inspection of the SCS is usually undertaken by the Kenya Wildlife Service (KWS) and Pan-African Sanctuaries Alliance (PASA) to ensure adherence to animal welfare provisions. Our sampling protocols were based on guidelines set by PASA Veterinary division and the KWS wild animals’ immobilization and treatment protocols, 2006.

### Study Area

The Sweet waters Chimpanzee Sanctuary (SCS) consist of a 1.01 km^2^ enclosure within the 364 km^2^ area of Ol Pejeta Conservancy, which lies west of Nanyuki in the Laikipia plateau at Latitude 0° 3′ North and Longitude 36° 56′ East. The vegetation at the Sanctuary comprises a mosaic of grasslands, *Acacia* woodlands, *Euclea* shrubs and riverine woodlands. The Sanctuary is also inhabited by native wild mammalian species such as antelopes, non-human primates, rodents and warthogs.

### The Chimpanzee Population

The Sanctuary was established to provide a life-long refuge to chimpanzees rescued from illegal trade, injured or orphaned by poaching or displaced from their natural habitats. Most chimpanzees in the Sanctuary were rescued from illegal captivity or confiscated from illegal wildlife trafficking. The Sanctuary rehabilitates and cares for these chimpanzees for the remainder of their lives in the safety of a vast natural enclosure.

At the time of the health survey, the Sweetwaters Chimpanzee Sanctuary maintained 42 chimpanzees in a square kilometer enclosure divided into the Eastern and Western sections by the Ewaso Ngiro River. The Western section (group B) maintained 14 chimpanzees (9 females and 5 males), while the Eastern section (group A) had 28 chimpanzees (14 females and 14 males). The chimpanzees have access to a natural environment within the Sanctuary which consists of bushes of *Euclea* and *Acacia* trees interspersed with open grassland areas. Chimpanzees roam freely within their sections of the natural environment during the day, but at night, they use indoor sleeping pens, approximately 3.5×4.6×4.2 meters in size, each shared by four individual chimpanzees. The chimpanzees at the Sanctuary are fed locally available fresh food of the season, consisting of fruits (e.g. bananas, oranges, mangoes and avocado), vegetables (e.g. carrots, tomatoes, green maize cob and collard greens), starch sources, (cooked maize meal, Irish and sweet potatoes) and occasional proteins sources (eggs, peanuts and kidney beans). The food is provided to the chimpanzees three times a day with additional food scattered across outdoor enclosures to encourage exploration and foraging outside as a part of behavioral enrichment. Animal welfare provisions include daily access to the big enclosures with behavioral enrichment materials including termite mounds, toys, and climbing sheds. In addition to routine health reviews, chimpanzees in this population are also assessed for gastric parasites and dewormed at three months’ interval [73].

### Blood Sample Collection and Processing

Blood samples were collected and morphometric measurements taken as part of routine care for this semi captive chimpanzee population. Blood samples and morphometric data were collected from 41 (Females, n = 21 and males, n = 20) apparently healthy individuals that had fasted without food or water for 12 hours. The animals were anaesthetized by intramuscular injection of a combination of Ketamine hydrochloride (Agrar, Holland BV) and Medetomidine hydrochloride (Pfizer laboratories (Pty) Limited, Sandton, South Africa) at a dosage of 4 mg/kg and 0.04 mg/kg body weight, respectively. From each individual chimpanzee sampled during this study, we collected 3 ml of venous blood from the femoral vein into a plain tube and another 3 ml into an EDTA-coated vacutainer for serum biochemistry and haematology respectively. Blood in EDTA was mixed by gently flipping the tube several times by hand. All samples were placed in a cool box and transported to Nanyuki Cottage hospital laboratory where they were analyzed within four hours of collection. Physical examination involved measuring cardio-respiratory and morphometric parameters. The following morphometric parameters were collected; body mass (kilograms), crown to rump length, chest and abdominal circumference, and fore and hind limb length. Reversal of Medetomidine hydrochloride was achieved 45 minutes after recumbency, using intramuscular injection of 0.04 mg/kg body weight of Atipamazole hydrochloride (Kyron Laboratories (Pty) Ltd, South Africa).

In the laboratory, non-EDTA blood samples were centrifuged at 3000 rpm for 15 minutes in order to separate sera followed by serum chemistry analyses. EDTA blood samples were subjected to automated hematological analyses using Cell Counter Analyzer MS9-5H (Melet Schloensing Laboratories). Hematology assays included complete cell counts, differential counts and other hematological parameters of clinical interest. Complete blood cell counts were determined for WBCs (leukocytes), RBCs (erythrocytes) and platelets (thrombocytes) and cell counts were expressed as number of cells per cubic milliliter of blood. In addition, the differential count of WBC components particularly, lymphocytes, neutrophils/granulocytes, monocytes, eosinophil and basophils were determined and the results presented as a percent of each WBC component from the total WBC count for each individual. Other hematological assays of clinical interest that were quantified include RBC characteristics such as mean corpuscular volume (MCV), hematocrit (Hct), mean corpuscular hemoglobin (MCH), mean corpuscular hemoglobin concentration (MCHC), hemoglobin (Hgb), red blood cell distribution width (RDW-SD), and red blood cell microparticles (µRBC) as a percent of total red blood cell count. Finally, important clinical characteristics of platelets were also quantified including, mean platelet volume (MPV), plateletcrit (Pct), platelet distribution width (PDW), and platelet microparticles (µPLT) expressed as a percent of the total platelet component.

### Estimation of Age in Chimpanzees

Ages of individual animals received at the Sweetwaters Chimpanzee Sanctuary were estimated based on the assessment of the dental development [74], [75], and the records obtained by the Sanctuary during the acquisition process. Exact ages were known from individual records for a few animals born at the Sanctuary.

### Estimation of Body Mass Index (BMI)

BMI for each chimpanzee was calculated by dividing body mass in kilograms by the square of the Crown to Rump length in meters. This is a standard formulae for determining BMI in non-human primates and has been successfully used to determine BMI of captive chimpanzees, baboons and macaques [8], [76].

### Statistical Analyses

Because our dependent variables were counts consisting of non-negative integers, we used a generalized linear mixed effect model frame work (GLMM) with a Poisson log-link function. Our dependent variables included proportion counts (e.g. platelet microparticles, neutrophils, lymphocytes, neutrophil to lymphocyte ratio, and red blood cell microparticles) and absolute counts (e.g. WBCs, platelets and red blood cells). For counts in which we were interested in their proportions, we used the denominator of the proportion as the offset in our GLMM analysis; for neutrophils and lymphocytes, we used the absolute WBC count as the offset; for the neutrophil to lymphocyte ratio, we used the neutrophil counts as our response variable and the lymphocyte count as the offset. Finally, for platelet microparticle and red blood cell microparticle proportions, we used the absolute platelet count and RBC count as offsets respectively. Our predictor variables (fixed effects) included age, BMI, and sex. Age and BMI where centered at the mean in all statistical analyses. Our sample sizes reduced to 20 males and 19 females after excluding individuals with missing data from our multivariate model analyses. We used chimpanzee group ID as a random factor in all models where it did explain substantial variance based on model selection using Bayesian information criteria (BIC). We compared our best model in each analysis to a simple intercept only model using ANOVA and the probability values for these tests are provided in the results.

We ran different linear models for males and females. We also combined the datasets for males and females and included sex, age, BMI and their interaction in order to test for differences in the mean and slope of the dependent variable with respect to the predictor variables between males and females. In all models involving sex as the independent variable, the female sex was taken as baseline; hence all coefficients related to sex are interpreted as the effect of being male relative to being female on the dependent variable.

## Results

### Age, BMI and Blood Measurements in the Study Population

Male ages in our sample of 19 chimpanzees ranged from 7 to 31 years with a mean ±1 standard deviation of 15.84±7.83 years. We had data from 20 females ranging in age from 3 to 28 with a mean age of 16.5±7.98. BMI among males ranged from 71.17 to 142.62 and the mean BMI ±1 standard deviation was 110.28±18.42. In females, BMI ranged from 58.36 to 239.20 and mean BMI ±1 standard deviation was 115.00±35.12. The mean count for RBCs, WBCs, platelets, lymphocytes and neutrophils were within expected variation of normal values established for captive chimpanzees (Table S1). All cell counts are expressed as ×10^3^ cells per cubic milliliter.

### White Blood Cells

Total WBC count declined with increase in age and BMI in males (P<0.001, Table 1). In females, the WBC count also declined with increase in age and BMI, however, there was a significant synergistic interaction between BMI and age in females (P<0.001, Table 1). WBC count from male and female chimpanzees combined was predicted by age, sex, BMI, and the interaction between sex, BMI and age (P<0.001, Table 1). Specifically, the WBC count, decreased with increase in age, BMI and the interaction between age and BMI. This joined male and female model also indicated that the decline in WBC counts was higher in females than in males.

**Table 1:** Coefficients showing the influence of age, BMI and sex on WBCs.

Variables	Coefficient	Standard error	Z statistic	Probability
**Males**				
*Intercept*	6.885	0.166	41.57	<0.001
*Age*	−0.101	0.009	−11.73	<0.001
*BMI*	−0.041	0.009	−4.77	<0.001
**Females**				
*Intercept*	6.965	0.046	151.19	<0.001
*Age*	−0.191	0.008	−25.06	<0.001
*BMI*	−0.119	0.009	−13.40	<0.001
*Age:BMI*	−0.058	0.009	−6.16	<0.001
**Males & Females combined**				
*Intercept*	6.978	0.121	57.73	<0.001
*Age*	−0.177	0.007	−25.18	<0.001
*BMI*	−0.093	0.007	−13.22	<0.001
*Sex*	−0.099	0.171	−0.58	0.561
*Age:BMI*	−0.043	0.007	−6.22	<0.001
*Age:Sex*	0.076	0.013	6.02	<0.001
*BMI:Sex*	0.034	0.015	2.26	0.024
*Age:BMI:Sex*	0.051	0.016	3.28	0.001

### Neutrophils and Lymphocytes

NLR increased in older ages and in high-BMI males (P<0.001, Table 2). BMI interacted with age such that in males, older individuals with a higher BMI had a decline in the NLR. In females NLR increased with age, but weakly declined with increase in BMI (P<0.001, Table 2). In females however the positive interaction between age and BMI suggest that NLR synergistically increased with age and with increase in BMI in older individuals. When we combined males and female data, our best model indicated that NLR was predicted by age, BMI and by a strong interaction between age, BMI and sex (P<0.001, Table 2). Specifically, NLR increased in older and higher BMI females relative to males.

**Table 2:** Coefficients showing the influence of age, BMI and sex on NLR.

Variables	Coefficient	Standard error	Z statistic	Probability
**Males**				
*Intercept*	0.979	0.298	3.28	0.001
*Age*	0.243	0.012	20.04	<0.001
*BMI*	0.116	0.011	10.45	<0.001
*Age:BMI*	−0.036	0.012	−3.11	0.002
**Females**				
*Intercept*	0.800	0.042	19.04	<0.001
*Age*	0.051	0.010	5.17	<0.001
*BMI*	−0.094	0.011	−8.35	<0.001
*Age:BMI*	0.210	0.012	17.92	<0.001
**Males & Females combined**				
*Intercept*	0.808	0.213	3.79	<0.001
*Age*	0.032	0.009	3.55	<0.001
*BMI*	−0.077	0.009	−8.65	<0.001
*Sex*	0.192	0.301	0.64	0.523
*Age:BMI*	0.157	0.009	18.03	<0.001
*Age:Sex*	0.212	0.016	13.23	<0.001
*BMI:Sex*	0.249	0.019	13.24	<0.001
*Age:BMI:Sex*	−0.212	0.020	−10.71	<0.001

The proportion of the neutrophils constituting the WBC count increased with increase in age among males (P<0.001, Table 3). There was a weak interaction between age and BMI among males such that individuals that were older and had a higher BMI had a slight decline in neutrophils in relation to the total WBC count. Among females however, the proportion of the neutrophil component of the WBCs was higher in older chimpanzees and in chimpanzees with a high BMI, but this pattern was largely driven by the interaction between age and BMI (P<0.001, Table 3). In a combined male and female dataset, the proportion of neutrophils increased at older age and at high BMI in both males and females. This increase in NLR was however, less in males compared to females (P<0.001, Table 3).

**Table 3:** Model coefficients for the influence of age, BMI on the proportion of neutrophils.

Variables	Coefficient	Standard error	Z statistic	Probability
**Males**				
*Intercept*	−0.507	0.073	−6.92	<0.001
*Age*	0.074	0.013	5.65	<0.001
*BMI*	0.033	0.011	2.93	0.003
*Age:BMI*	−0.025	0.012	−2.09	0.036
**Females**				
*Intercept*	−0.531	0.009	−56.51	<0.001
*Age*	0.018	0.010	1.82	0.069
*BMI*	−0.010	0.011	−0.95	0.343
*Age:BMI*	0.067	0.011	5.89	<0.001
**Males & Females combined**				
*Intercept*	−0.527	0.052	−10.21	<0.001
*Age*	0.011	0.009	1.21	0.226
*BMI*	−0.011	0.009	−1.19	0.233
*Sex*	0.026	0.073	0.36	0.720
*Age:BMI*	0.052	0.009	5.96	<0.001
*Age:Sex*	0.062	0.017	3.60	<0.001
*BMI:Sex*	0.061	0.019	3.18	0.001
*Age:BMI:Sex*	−0.090	0.021	−4.38	<0.001

The proportion of lymphocytes which constitute the WBC count declined with increase in age and BMI in males (P<0.001, Table 4), but not in females (P = 0.357, Table 4). When we combined data to test for a sex effect males compared to females had a higher initial proportion of lymphocytes. The proportion of lymphocytes however, declined rapidly in response to increase in BMI and increase in age in males compared to females (P<0.001, Table 4).

**Table 4:** Coefficients showing the influence of age, BMI and sex on the proportion of lymphocytes.

Variables	Coefficient	Standard error	Z statistic	Probability
**Males**				
*Intercept*	−1.485	0.218	−6.81	<0.001
*Age*	−0.190	0.017	−10.94	<0.001
*BMI*	−0.093	0.016	−5.74	<0.001
**Females**				
*Intercept*	−1.326	0.014	−96.13	<0.001
*Age*	0.011	0.014	0.78	0.433
*BMI*	0.018	0.017	1.08	0.281
**Males & Females combined**				
*Intercept*	−1.332	0.157	−8.49	<0.001
*Age*	−0.013	0.014	−0.94	0.349
*BMI*	0.042	0.015	2.86	0.004
*Sex*	−0.165	0.222	−0.74	0.457
*Age:BMI*	−0.108	0.015	−7.45	<0.001
*Age:Sex*	−0.172	0.027	−6.33	<0.001
*BMI:Sex*	−0.177	0.030	−5.96	<0.001
*Age:BMI:Sex*	0.128	0.032	4.03	<0.001

### Platelets

Platelet counts were negatively correlated with age and BMI such that it was lower in older males and in males with a higher BMI than among younger males and males with a lower BMI. There was a weak and negative interaction between BMI and age indicating that the influence of BMI and age on platelets is multiplicative in males (P<0.001, Table 5). Similarly, the platelet count in females was negatively correlated with age and BMI (P<0.001, Table 5). When we combined data from both sexes and tested for a sex effect, our best model included BMI, age, sex and their interactions as predictors. Specifically, platelet counts declined with increase in age and BMI (P<0.001, Table 5). Females had a higher platelet count than males on average, but the decline in platelets with age and BMI was higher in males than in females.

**Table 5:** Coefficients showing the influence of age, BMI and sex on blood platelets.

Variables	Coefficient	Standard error	Z statistic	Probability
**Males**				
*Intercept*	5.588	0.059	94.34	<0.001
*Age*	−0.208	0.018	−11.43	<0.001
*BMI*	−0.114	0.016	−7.23	<0.001
*Age:BMI*	−0.059	0.016	−3.63	<0.001
**Females**				
*Intercept*	5.652	0.043	131.99	<0.001
*Age*	−0.072	0.014	−5.31	<0.001
*BMI*	−0.202	0.017	−12.08	<0.001
**Males & Females combined**				
*Intercept*	5.669	0.051	111.39	<0.001
*Age*	−0.068	0.014	−5.01	<0.001
*BMI*	−0.159	0.013	−11.79	<0.001
*Sex*	−0.102	0.072	−1.40	0.161
*Age:BMI*	0.002	0.013	0.17	0.863
*Age:Sex*	−0.155	0.024	−6.38	<0.001
*BMI:Sex*	−0.013	0.027	−0.47	0.635
*Age:BMI:Sex*	−0.093	0.028	−3.27	0.001

### Platelet Microparticles

The proportion of µPLT that constitute the platelet component of the blood was positively correlated with age and BMI (i.e. µPLT were higher in older and in high-BMI individuals than in younger or low-BMI individuals) in separate analysis including only males (P<0.001, Table 6) and only females (P<0.001, Table 6). In the analysis including only males however, there was a negative interaction between BMI and age indicating that at older ages and at high BMI the proportion of µPLT declined. When males and females were combined in a single analysis, the best model predicting increase in µPLT included sex, age, BMI and the interaction between age, BMI and sex as predictor variables. These results indicate that males have a higher baseline level of µPLT than females and that the rate of increase in µPLT is lower in females compared to males (P<0.001, Table 6).

**Table 6:** Coefficients showing the influence of age, BMI and sex on platelet microparticles.

Variables	Coefficient	Standard error	Z statistic	Probability
**Males**				
*Intercept*	−1.730	0.035	−48.79	<0.001
*Age*	0.209	0.039	5.34	<0.001
*BMI*	0.202	0.034	5.94	<0.001
*Age:BMI*	−0.102	0.035	−2.96	0.003
**Females**				
*Intercept*	−1.712	0.032	−53.93	<0.001
*Age*	0.096	0.031	3.08	0.002
*BMI*	0.112	0.033	3.42	0.001
**Males & Females combined**				
*Intercept*	−1.723	0.032	−53.45	<0.001
*Age*	0.091	0.031	2.93	0.003
*BMI*	0.089	0.031	2.83	0.005
*Sex*	0.025	0.048	0.53	0.595
*Age:BMI*	−0.001	0.030	−0.05	0.962
*Age:Sex*	0.109	0.052	2.07	0.038
*BMI:Sex*	0.212	0.060	3.54	<0.001
*Age:BMI:Sex*	−0.156	0.061	−2.55	0.011

### Red Blood Cells

RBC counts were lower in older males and in males with high BMI than in young males or males with low BMI. BMI influenced the RBC count through its interaction with age in males (P<0.001, Table S2). In contrast, age and BMI did not influence RBC counts in females (P = 0.830, Table S2). When we combined female and male data to test for a sex effect, we found an interaction between age, sex and BMI, which indicated that males had a higher initial RBC count and a more rapid decline in red blood cell count in older ages and in individuals with a higher BMI compared to females (P<0.001, Table S2).

### Red Blood Cell Microparticles (µRBC)

µRBC was not predicted by age and BMI in separate analyses for males only, females only, or males and females combined (Table S3). The best models for both female and combined male and female data was the intercept only model whereas the best model for male data contained age, BMI and the interaction between age and BMI (Table S3).

## Discussion

Older age, elevated BMI status and male sex in chimpanzees were positively correlated with biomarkers of inflammation risk established for humans such as NLR, platelet microparticles and neutrophils, but negatively correlated with total platelets and total WBCs. RBCs were negatively correlated with age and BMI in male but not in female chimpanzees. In contrast, µRBC were not related to either age or BMI. We discuss each of these findings below.

First, NLR was higher in older individuals compared to younger individuals in both males and females. This difference was mostly driven by a higher proportion of neutrophils in older than in younger individuals and by the decline in lymphocytes with age in males. Increasing NLR with age has recently been observed in silver foxes, *Vulpes vulpes* and raccoon dogs, *Nyctereutes procyonoides*, [77]. The positive correlation between neutrophils and age and the negative correlation between lymphocyte counts and age support findings from captive chimpanzees elsewhere [78]. Increase in age is correlated with increased production of reactive oxygen species (ROS) [79], [80] that damage tissues resulting in neutrophil activation. Activated neutrophils in turn cause further inflammation, while making inflamed tissues non-responsive to negative feedback mechanisms that modulate inflammation [81]. In addition, ageing is marked by increased secretion of pro-inflammatory cytokines or proteins such as granulocyte-macrophage colony stimulating factor (GM-CSF) by senescing cells which not only stimulates the production and differentiation of neutrophils [82] but may also enhance their longevity. For instance, pro-inflammatory cytokines are known to suppress neutrophil apoptosis, thereby exacerbating the inflammatory effects of neutrophils [83], [84]. Elevated ROS and pro-inflammatory cytokine production contribute to elevated proportion of neutrophils relative to other WBC components.

The positive correlation between BMI and NLR observed in this study is supported by similar observations in humans [85], [86]. For example, Wang *et al.* observed an increase in NLR with increase in age, while Dixon *et al.* observed an increase in neutrophils compared to that of lymphocytes in response to increase in obesity [85], [86].

We also found a difference in mean NLR between males and females. Specifically, males had higher mean NLR than females. No studies that we are aware of have examined whether there is a sex difference in mean NLR in either humans or in non-human primates.

Second, the proportion of µPLT was positively correlated with age and BMI in both male and female chimpanzees. Although there is a dearth of information on the changes of µPLT with age in humans and in non-human primates, these observations are in agreement with increase of inflammation risks with age and obesity status in humans [16], [18], [87]. Increased platelet activation and elevated µPLT are driven by signals from various stimuli including fatty-acid-accumulation resulting from obesity, reactive oxygen species (e.g. hydrogen peroxide) resulting from oxidative stress associated with the ageing process and obesity [16], [88]–[90]. The positive correlation between BMI and µPLT we observed in chimpanzees are similar to those in humans. Murakami *et al.* found that µPLT values were elevated in the obese individuals compared with non-obese individuals. Murakami *et al.* also documented a significant reduction in µPLT among the obese group after weight loss [90]. Additionally, male chimpanzees in our study had a higher baseline µPLT count as well as an increasing µPLT count with age than females. A higher concentration of µPLT among males compared to females has been observed in humans [91]. This could result from the fact that the release of µPLT in females is suppressed by estrogen [92]. For example, a low level of estrogen in the blood of females is associated with increased micro vesiculation and microparticle release [92].

Third, we observed parallels between the influence of older age and higher BMI on declining immune function. Specifically, WBC and platelet counts were low in older individuals. These results support observations from other studies in humans and chimpanzee which have shown declines in platelet and WBCs as age increases [93]–[96]. In contrast to human studies, WBC count was negatively correlated with BMI in chimpanzees [30], [85], [97]. Similarly, platelet counts were also negatively correlated with BMI in contrast to the findings from human studies [98], [99]. These results which contrast with findings from human studies should be interpreted with caution as our captive chimpanzee population had lower BMI compared to published BMI from other populations [8]. The absence of a positive relationship between BMI and WBC or platelets in this study may indicate the presence of a non- linear correlation between BMI and WBC or platelets and a low BMI threshold for detecting any effect of BMI on these blood parameters in our study population.

Fourth, we found no relationship between µRBC and age or BMI in either male or female chimpanzees, but we observed a significant sex dependent decline in RBC counts in older males but not in females. No studies to our knowledge have examined the influence of age on µRBC; rather, studies have examined the relationship between RBC and age. These studies observed a decline in red blood cell count in males and no decline in females with respect to increasing age in some human populations [100], [101] and in captive chimpanzee populations [78], [102]. Other studies on captive chimpanzee populations have demonstrated no age effect but a significant sex effect on RBC count [78], [94], [96], [102].

## Conclusion

Our results support the observation that ageing and obesity are accompanied by chronic low-grade inflammatory state because higher values of platelet microparticles, neutrophils and the neutrophil to lymphocyte ratio -known biomarkers of inflammation in humans- were generally higher in older and higher BMI individuals [18], [99], [103], [104]. Additionally, there was a positive sex by age interaction on inflammation risk, with older males more at risk than older females. These results suggest that in chimpanzees like in humans, there exist a strong link between disease risk and age, sex and BMI.

The positive relationship we found between BMI and inflammatory disease risk in chimpanzees suggests that the management of BMI in captive chimpanzees is critical to their health, welfare and longevity as demonstrated by studies on calorie restriction in other primates [105]–[109]. Management should combine husbandry practices that enhance play and exercise and design diet regimes that reduce obesity and maintain normal BMI levels. Currently, there is no characterization of BMI values considered normal or obese for management of the health of chimpanzees in captivity. Markers for inflammation risk assessed in this study will be combined with health history records to designate acceptable BMI records in future studies of this population.

## Supporting Information

Table S1 Comparison of selected hematological parameters from the Sweetwaters Sanctuary with expected normal variation for captive chimpanzees.
(DOCX)

Table S2 Coefficients showing the influence of age, BMI and sex on RBC.
(DOCX)

Table S3 Coefficients showing the influence of age, BMI and sex on µRBC.
(DOCX)

Checklist S1 (DOC)
