# Supplementary material for: The Influence of Body Mass Index, Age and Sex on Inflammatory Disease Risk in Semi-Captive Chimpanzees
Source: PLoS One. 2014 Aug 14;9(8):e104602. doi: 10.1371/journal.pone.0104602 (PMC4133249; doi:10.1371/journal.pone.0104602)
Supplement: Table S1 — Comparison of selected hematological parameters from the Sweetwaters Sanctuary with expected normal variation for captive chimpanzees. (DOCX) [file pone.0104602.s001.docx]

**Table S1**: Comparison of selected hematological parameters from the Sweetwaters Sanctuary with expected normal variation for captive chimpanzees

|  | Male  Mean ± SE | Normal Male variation  Mean±2SD | Female  Mean ± SE | | Normal Female variation  Mean±2SD | |
| --- | --- | --- | --- | --- | --- | --- |
| *Age (years)* | 15.95 ± 1.71 | - | 16.21 ± 1.9 | | - | |
| *BMI* | 111.25 ± 4.13 | - | 116.81 ± 8.07 | | - | |
| *Platelets (PLT)* | 265.8 ± 14.24 | 195.6 ± 96.4 | 296 ± 22.42 | | 230.1 ± 132.7 | |
| *NLR* | 2.66 ± 0.62 | 2.08 | 2.54 ± 0.41 | | 1.27 | |
| *WBC* (x10^3^/mm^3^) | 8.89 ± 0.71 | 9.2 ±6.9 | 11.00 ± 1.06 | 9.1 ± 6.3 | |  |
| *RBC* (x10^3^/mm^3^) | 5.38 ± 0.13 | 5.5 ±0.7 | 5.10 ± 0.104 | | 5.1 ± 0.9 | |
| *Neutrophils* (x10^3^/mm^3^) | 5.24 ± 0.64 | 5.4 ±5.8 | 6.53 ± 0.78 | | 4.5 ± 8.5 | |
| *Lymphocytes* (x10^3^/mm^3^) | 2.39 ± 0.19 | 2.6 ±1.8 | 2.91 ± 0.28 | | 3.7 ± 3.1 | |
| *% µPLT* | 48.70 ± 3.96 | - | 52.47 ± 1.86 | | - | |
| *% µRBC* | 3.45 ± 0.63 | - | 2.95 ± 0.21 | | - | |

NLR, is the neutrophil to lymphocyte ratio, WBC is the white blood cell count, RBC is the red blood cell count, % µPLT is percent of platelet microparticles and % µRBC, is percent of RBC microparticles. Normal values are based on adult values provided by Howell, et al., (2003 )

**References**

Howell S, Hoffman K, Bartel L, Schwandt M, Morris J, Fritz J, 2003. Normal hematologic and serum clinical chemistry values for captive chimpanzees (*Pan troglodytes*). Comparative Medicine 53:413-423.

Ihrig M, Tassinary LG, Bernacky B, Keeling ME, 2001. Hematologic and serum biochemical reference intervals for the chimpanzee (*Pan troglodytes*) categorized by age and sex. Comparative Medicine 51:30-37.
