# Supplementary material for: The Influence of Body Mass Index, Age and Sex on Inflammatory Disease Risk in Semi-Captive Chimpanzees
Source: PLoS One. 2014 Aug 14;9(8):e104602. doi: 10.1371/journal.pone.0104602 (PMC4133249; doi:10.1371/journal.pone.0104602)
Supplement: Table S2 — Coefficients showing the influence of age, BMI and sex on RBC. (DOCX) [file pone.0104602.s002.docx]

**Table S2**: Coefficients showing the influence of age, BMI and sex on RBC

|  | Estimate | Std. Error | Z. Value | P. Value |
| --- | --- | --- | --- | --- |
| Males |  |  |  |  |
| *Intercept* | 6.268 | 0.010 | 604.82 | <0.001 |
| *Age* | -0.042 | 0.012 | -3.58 | <0.001 |
| *BMI* | -0.011 | 0.010 | -1.07 | 0.285 |
| *Age:BMI* | -0.058 | 0.011 | -5.32 | <0.001 |
|  |  |  |  |  |
| Females |  |  |  |  |
| *Intercept* | 6.238 | 0.010 | 603.02 | <0.001 |
| *Age* | -0.002 | 0.011 | -0.20 | 0.843 |
| *BMI* | 0.005 | 0.013 | 0.36 | 0.721 |
| *Age:BMI* | -0.021 | 0.013 | -1.57 | 0.117 |
|  |  |  |  |  |
| Males & Females |  |  |  |  |
| *Intercept* | 6.238 | 0.010 | 606.21 | <0.001 |
| *Age* | 0.000 | 0.010 | -0.05 | 0.963 |
| *BMI* | 0.004 | 0.010 | 0.38 | 0.704 |
| *Sex* | 0.028 | 0.015 | 1.90 | 0.058 |
| *Age:BMI* | -0.015 | 0.010 | -1.57 | 0.117 |
| *Age:Sex* | -0.051 | 0.016 | -3.13 | 0.002 |
| *BMI:Sex* | -0.022 | 0.019 | -1.18 | 0.238 |
| *Age:BMI:Sex* | -0.074 | 0.019 | -3.80 | <0.001 |
