# Supplementary material for: The Influence of Body Mass Index, Age and Sex on Inflammatory Disease Risk in Semi-Captive Chimpanzees
Source: PLoS One. 2014 Aug 14;9(8):e104602. doi: 10.1371/journal.pone.0104602 (PMC4133249; doi:10.1371/journal.pone.0104602)
Supplement: Table S3 — Coefficients showing the influence of age, BMI and sex on µRBC. (DOCX) [file pone.0104602.s003.docx]

**Table S3**: Coefficients showing the influence of age, BMI and sex on **µRBC**

| Variables | Coefficient | Standard Error | Z statistic | Probability |
| --- | --- | --- | --- | --- |
| Males |  |  |  |  |
| *Intercept* | -5.03 | 0.129 | -38.970 | <0.001 |
| *Age* | -0.102 | 0.158 | -0.640 | 0.521 |
| *BMI* | -0.246 | 0.137 | -1.800 | 0.072 |
|  |  |  |  |  |
| Females |  |  |  |  |
| *Intercept* | -5.144 | 0.135 | -38.090 | <0.001 |
| *Age* | 0.019 | 0.143 | 0.130 | 0.890 |
| *BMI* | -0.005 | 0.181 | -0.030 | 0.980 |
|  |  |  |  |  |
| Males & Females |  |  |  |  |
| *Intercept* | -5.144 | 0.134 | -38.300 | <0.001 |
| *Age* | 0.026 | 0.133 | 0.190 | 0.846 |
| *BMI* | -0.002 | 0.144 | -0.020 | 0.987 |
| *Sex* | 0.078 | 0.192 | 0.40 | 0.686 |
| *Age:BMI* | -0.075 | 0.140 | -0.540 | 0.590 |
| *Age:Sex* | -0.087 | 0.222 | -0.390 | 0.694 |
| *BMI:Sex* | -0.359 | 0.251 | -1.430 | 0.152 |
| *Age:BMI:Sex* | 0.516 | 0.280 | 1.850 | 0.065 |
